# QTL mapping of winter dormancy and associated traits in two switchgrass pseudo-F1 populations: lowland x lowland and lowland x upland

**DOI:** 10.1186/s12870-020-02714-8

**Published:** 2020-11-30

**Authors:** Rasyidah M. Razar, Ali Missaoui

**Affiliations:** Institute of Plant Breeding Genetics and Genomics, and Department of Crop and Soil Sciences, University of Georgia, 30602 Athens, GA USA

**Keywords:** QTL mapping, Switchgrass, Winter dormancy, Spring emergence, Flowering date

## Abstract

**Background:**

Switchgrass (*Panicum virgatum*) undergoes winter dormancy by sensing photoperiod and temperature changes. It transitions to winter dormancy in early fall following at the end of reproduction and exits dormancy in the spring. The duration of the growing season affects the accumulation of biomass and yield. In this study, we conducted QTL mapping of winter dormancy measured by fall regrowth height (FRH) and normalized difference vegetation index (NDVI), spring emergence (SE), and flowering date (FD) in two bi-parental pseudo-F1 populations derived from crosses between the lowland AP13 with the lowland B6 (AB) with 285 progenies, and the lowland B6 with the upland VS16 (BV) with 227 progenies.

**Results:**

We identified 18 QTLs for FRH, 18 QTLs for NDVI, 21 QTLs for SE, and 30 QTLs for FD. The percent variance explained by these QTLs ranged between 4.21–23.27% for FRH, 4.47–24.06% for NDVI, 4.35–32.77% for SE, and 4.61–29.74% for FD. A higher number of QTL was discovered in the BV population, suggesting more variants in the lowland x upland population contributing to the expression of seasonal dormancy underlying traits. We identified 9 regions of colocalized QTL with possible pleiotropic gene action. The positive correlation between FRH or NDVI with dry biomass weight suggests that winter dormancy duration could affect switchgrass biomass yield. The medium to high heritability levels of FRH (0.55–0.64 H^2^) and NDVI (0.30–0.61 H^2^) implies the possibility of using the traits for indirect selection for biomass yield.

**Conclusion:**

Markers found within the significant QTL interval can serve as genomic resources for breeding non-dormant and semi-dormant switchgrass cultivars for the southern regions, where growers can benefit from the longer production season.

**Supplementary Information:**

The online version contains supplementary material available at 10.1186/s12870-020-02714-8.

## Background

Switchgrass, *Panicum virgatum* L. is a C4 warm season grass native to most of North America, ranging from southern Canada to northern Mexico [1]. It is predominantly cross-pollinated due to gametophytic self-incompatibility [2]. Switchgrass germplasm is divided taxonomically into two major ecotypes, upland and lowland [1]. The basic chromosome number of switchgrass is x = 9. Lowland ecotypes are mostly tetraploid (2n = 4x = 36 chromosomes) and, rarely, octoploid (2n = 8x = 72), while upland ecotypes can be tetraploid or octoploid [3]. Switchgrass uses include animal grazing, soil conservation, landscaping, and more recently as biofuel feedstock [1]. Switchgrass has been selected as a herbaceous model species by the U.S. Department of Energy (DOE) Biofuel Feedstock Development Program (BFDP) in 1991 for biofuel production [4]. Its suitability for planting in marginal land unsuitable for row crops makes it the right species for biofuel [5].

The taxonomic division of switchgrass is caused by the difference in latitudinal adaptation which results in phenotypic differences accounted by the prevalent photoperiods and temperatures along the latitudes of their adaptive environments [1, 6–10]. Upland ecotypes are mostly found at the northern latitudes where they evolved adaptation traits for a short growing season and tolerance to cold winter temperatures [11]. Lowland ecotypes are found at the southern latitudes and are adapted to a longer growing season but are sensitive to cold temperatures. Lowland ecotypes differ from the upland ecotypes in that they are taller, have fewer and larger tillers, longer and wider leaf blades, and thicker stems. Upland ecotypes may flower as early as late June or early July while lowland ecotypes flower as late as mid-October [12].

Casler, et al. [6] measured the ground cover after spring emergence and in fall harvest and found lower values for both ecotypes planted at their non-adapted locations, suggesting planting at different latitudes only favors the adapted ecotypes [6]. Because of the different adaptability to the photoperiod and temperature, upland ecotypes are naturally found in the northern hardiness zones of 2–7 while lowland ecotypes can be found in the southern zones of 6–10 [11]. Casler [1] reported eight regional gene pools or cultivar deployment zones based on the adaptation of accessions to the regional photoperiod and temperature levels. These two factors largely affect switchgrass spring emergence, flowering time, cold and heat tolerance, and the onset of winter dormancy [1, 6, 13].

Switchgrass like other warm-season perennial grasses responds to photoperiod changes and this sensitivity is genetically controlled. They depend on the photoperiod cues to initiate growth in favorable conditions while going dormant when conditions are unfavorable [11]. Under longer photoperiod, the growth of switchgrass is much greater and flowering is delayed [7, 8]. Van Esbroeck et al. [8] found a delay in panicle emergence and a longer duration of panicle exertion in the northern switchgrass cultivar, Cave-in-Rock, under longer photoperiod. The delay was thought to be associated with an increase in the phyllocron, the intervening period between the sequential emergences of leaves on the main stem of a plant. The study confirmed that switchgrass has a facultative short-day response, flowering earlier under short days. Northern populations of switchgrass that are adapted to longer daily photoperiod in summer tend to flower early and mature more rapidly when planted at southern latitudes [14]. The opposite happened when southern populations were planted at northern latitudes; they flowered later and remained vegetative longer [14].

Temperature is another factor controlling switchgrass growth. Metabolic pathways such as photosynthesis, respiration, and growth processes are catalyzed by enzymes which activities are affected by temperature. An optimum switchgrass growth was determined at a moderately high temperature between 25 to 30 °C but decreased at a higher temperature of 37.6 °C [15] or a cooler temperature of 14 °C [16]. Freezing temperatures could also result in plant death. Evaluation of both switchgrass ecotypes in northern locations showed near-complete survival of upland ecotypes and near complete mortality of the lowland ecotypes due to the inadaptability to extreme cold temperatures [17].

Dormancy means a lack of visible growth [18, 19], an adaptive mechanism of plant species for survival in threatening environments [20]. An example of a harsh environment for plant growth is the cold weather in winter. Perennial plants undergo cyclical growth that responds to winter by terminating meristem growth and becoming insensitive to growth-promoting signals [21]. Exposure of non-dormant plants to cold temperatures may impair their physiological functions such as photosynthesis, cellular transport, and the ability to scavenge reactive oxygen species [22]. Freezing temperatures can also result in the formation of ice in the intracellular as well as extracellular spaces leading to damage to the cell structures and cell dehydration as a result of the non-availability of water for absorption [23].

The classification of dormancy is based on the primary physiological reactions causing dormancy [24] and the external trigger [25]. The terms ecodormancy, paradormancy, and endodormancy were suggested to describe three types of dormancy; ecodormancy is growth inhibition by temporary unfavorable environmental conditions; paradormancy is inhibition of growth by signals from distal organs; endodormancy is growth repression by internal signals [24, 26, 27]. Rohde and Bhalerao [21] defined endodormancy as “the inability to initiate growth from meristems under favorable conditions”. Preston and Sandve [23] defined endodormancy as physiological reactions to environmental factors, but these are not necessarily required for the initiation of dormancy and the plant can undergo dormancy even if environmental conditions are suitable for growth.

In switchgrass, endodormancy is triggered when the plant senses the changes in photoperiod and temperature before winter and consequently undergo senescence. During senescence, the leaf will endure systematic changes from cellular metabolism to degradation of cellular structures like chloroplasts, mitochondria, and nucleus [28]. The degradation of macromolecules enables the relocation of nutrients to the below-ground stems and storage organs [28]. The reserves stored in the switchgrass rhizomes and crowns will drive new tiller growth in the spring when the conditions are favorable for growth [23, 29, 30]. After spring emergence, warm-season grasses undergo a vegetative phase (leaf growth) followed by an elongation phase (internode length growth). The flowering phase will start in switchgrass when there are appropriate flowering signals perceived, switching the vegetative tiller meristems into reproductive tillers [29]. Flowering and seed developments are cues for tiller senescence in C4 perennial grasses and are possible factors in the induction of dormancy in the rhizomes [29].

Manipulating the onset and duration of winter dormancy is a potential strategy to increase biomass yield and seasonal distribution of switchgrass in the Southern USA where winters are mild. Currently, the selection of non-dormant plants is challenging because there is no dormancy rating system in place and phenotyping dormancy in segregating populations would require several seasons and multiple locations to have an accurate characterization. Understanding the genetic basis of seasonal dormancy in switchgrass and identifying quantitative trait loci (QTL) associated with the trait will enable the development of genomic resources for marker-assisted selection. In this study, QTL mapping of winter dormancy, spring emergence, and flowering date was conducted in two bi-parental pseudotestcross populations derived from the crosses between a non-dormant lowland tetraploid genotype, B6, and a dormant lowland tetraploid genotype, AP13 (AB population) and between B6 and a dormant upland tetraploid genotype, VS16 (BV population). This study would be the first to report on QTLs associated with winter dormancy in switchgrass with the intent to analyze the inheritance of the trait and its correlation with biomass yield.

## Methods

### Development of mapping populations

Two F1 populations consisting of 285 and 227 progenies derived from two crosses, AP13 x B6 (AB) and B6 x VS16 (BV), respectively, were produced in the greenhouse in 2015 and 2016. A non-dormant lowland tetraploid genotype B6 was crossed to a dormant lowland tetraploid genotype AP13 and a dormant upland tetraploid genotype VS16. The parents B6, AP13, and VS16 were selected from PI422001, “Alamo”, and “Summer” accessions, respectively. PI422001 accession was obtained from the Germplasm Resources Information Network (GRIN), while AP13 and VS16 were the parents of the mapping population AP13 x VS16 [31]. PI422001 is an accession from the USDA collection tracing back to the Stuart population originating from Florida. This genotype was identified as nondormant during the evaluation of a GWAS panel at the University of Georgia Plant Sciences Farm in Watkinsville in 2014.

Clones of the parental material were raised in the greenhouse for one year to validate their dormancy status and crosses were later conducted by pairing the parents in isolation in a separate greenhouse section to prevent cross-pollination from unidentified sources of switchgrass pollen. The plants were cross-pollinated by placing every two parents in close proximity. The seed produced from cross-pollinated plants were collected at maturity and dried at room temperature before undergoing pre-chilling treatment to break seed dormancy. In pre-chilling treatment, seeds were placed on a wet filter paper in a petri dish. The Petri dish was closed and sealed with parafilm then placed in a 4 °C refrigerator for two weeks. After that, the seeds were planted in flats for germination.

Seedlings were first genotyped using one polymorphic SSR marker before transferring them into bigger pots to ensure they are hybrids. The plants were later divided into three clones for replication. The parents used in the crosses were also divided into 3 clones and included in the field experiment. All 285 progenies and both parents from the AB population were transplanted to the field in April 2017. The BV population was planted in two steps. The first subset of the BV population comprising 66 progenies and both parents were transplanted in April 2017 (Planting date 1), while the second subset of 161 progenies was transplanted in May 2018 (Planting date 2). Both fields were located at the University of Georgia Iron horse plant sciences farm in Greene County, GA (33.73° N, − 83.30° W). The experimental design was a randomized complete block design with three replications. Plants were spaced apart by 3 ft (91.4 cm). The soil type is Cecil gravelly sandy loam. Field management included the preemergence and postemergence application of Pendimethalin and Atarazine herbicides before planting and irrigation of the field after planting. Herbicide application was repeated in the fall after harvest and in the spring before the emergence of switchgrass to prevent the germination of switchgrass seeds shattered from the previous season.

### Phenotypic data collection

Phenotypic data were collected in two growing seasons as described in [32]. The phenotypic traits were senescence level and plant regrowth height following clipping in early fall, spring emergence date, and flowering date. The extent of senescence and plant regrowth after clipping in early fall were considered indicators of dormancy initiation. Plants entering dormancy early will not have much regrowth after clipping and the foliage will start senescing as soon as photoperiod and temperature drop in late summer and early fall. The level of regrowth in the fall was measured at the height of 10 cm above the ground after clipping the plants on 31 August 2017 and on 24 September 2018 and letting them grow back for 4 weeks. The height of the new tillers was measured and termed fall regrowth height (FRH). Senescence was estimated using the normalized difference vegetation index (NDVI), where a high NDVI indicates a low senescence level [32]. NDVI has been used in numerous studies to estimate vegetation cover and the ‘greenness’ of plants [33–38]. NDVI was measured by scanning the plants at a fixed height of 80 cm above the ground using a GreenSeeker handheld crop sensor (http://www.trimble.com/Agriculture/gs-handheld.aspx),

The exit of plants from dormancy was determined by recording the date of spring emergence of new tillers starting between the first week of February and the end of April at the frequency of one observation every three days. The dates of spring emergence were converted to Julian calendar days, where the small Julian numbers indicate early regrowth. Flowering dates were estimated based on the date of panicle emergence on at least one stem and converted to Julian calendar days. Flowering date observations were recorded from early June until the end of July and were done every three days.

In addition to these four traits, the plants were harvested and the dry biomass weight was used to calculate the correlation between the four traits with biomass yield. First and second-year harvests were done on 24th September 2018 and 29th September 2019 using a Swift Machine forage plot harvester (Swift Machine & Welding Limited, Canada) for the 2018 harvest and a Wintersteiger Cibus F/S harvester (Wintersteiger Seedmech, Austria) for the 2019 harvest. Fresh biomass weight from the whole plant was measured after clipping. A sample from each plant was weighed and dried in a convection oven at 60 °C for 48 h, and then weighed again to determine the dry matter content. Whole-plant dry weight was calculated using each plant’s dry matter content multiplied by the fresh biomass weight.

### Statistical analysis of phenotypic data

Test for normality of phenotypic data was first conducted using PROC UNIVARIATE of SAS (SAS 9.4, SAS Institute Inc., Cary, NC, USA) using a Q-Q plot of residuals. Analysis of variance (ANOVA) was conducted to test the effects of genotype, year, and genotype by year interactions on the measured traits using PROC MIXED of SAS (SAS 9.4, SAS Institute Inc., Cary, NC, USA). Genotype, year, and genotype by year interaction were treated as fixed effects, while replications within years were treated as a random effect. Broad sense heritability for each trait was calculated using the formula:

$$ {H}^2=\frac{V_g}{V_g+\frac{V_{gy}}{y}+\frac{V_e}{ry}} $$

where V_g_ = Genotypic variance, V_gy_ = Genotype by year variance, V_e_ = Error variance, r = number of replications, and y = number of years. Variance components were generated using PROC VARCOMP of SAS (SAS 9.4, SAS Institute Inc., Cary, NC, USA) using the restricted maximum likelihood (REML) method. Correlation among traits and with biomass weight was estimated by Pearson’s correlation coefficient using PROC CORR of SAS (SAS 9.4, SAS Institute Inc., Cary, NC, USA). BLUP value of individual genotypes was used in the calculation of the correlation.

### QTL mapping

Three data files were used for QTL mapping: the genetic linkage map (linkage groups with ordered markers), the phenotypic data, and the marker profiles for the breeding population. SNP marker generation and construction of linkage maps were described in a separate study. In brief, SNP markers were developed using genotyping-by-sequencing methodology using two methylation-sensitive restriction enzymes: *PstI* and *MspI*. Two genetic maps were constructed for maternal and paternal recombination events in each population. Single-dose alleles (heterozygous in one parent and homozygous in the other parent) were used in marker grouping and ordering by JoinMap 5.0 [39].

Least square means for all phenotypic data were calculated separately for each year to avoid the effect of environment and genotype by environment interaction in QTL detection. For the BV population that was planted over two planting dates, we calculated the LS means separately for each planting date in each year. A mixed linear model was used to estimate the LS means where genotype was set as a fixed effect and replication as a random effect. We also pooled the data across years for AB and across years and planting dates for BV using Best Linear Unbiased Predictor (BLUP). BLUP shrinks the variance resulting from testing in different replicates and environments and subsequently generates the best predictive value for each genotype [40]. We used a mixed model to calculate BLUP where the random effect was set for genotype and a fixed effect for replication and year. For the BV population, the planting date was included as a fixed effect too. Piepho and Eckl [41] demonstrated how ryegrass varieties with different establishment dates within the same location were analyzed in a single mixed model. The trial date or planting date is treated as a blocking factor. Both BLUP and LS means were calculated using PROC MIXED in SAS 9.4 (SAS Institute Inc., Cary, NC, USA).

QTL mapping was carried out for four traits: FRH, NDVI, SE, and FD. We used both the LS means in each year and BLUP values to carry out the mapping using the Composite interval mapping (CIM) program in WinQTL Cartographer 2.5 [42]. Since this software is designed for inbred species with known linkage phase, the genetic marker of an outcrosser species needs to be adjusted to properly define the recombination interval. For this step, the progenies’ genotypes at a particular marker locus were inverted according to the linkage phase information given by the JoinMap data output. All markers’ linkage phase (in each LG) were standardized to one phase, i.e. all {1-} or {0-}, and the genotypes at the marker locus with different phases were inverted accordingly; heterozygous to homozygous and vice versa. QTL mapping was then carried out using this newly formed dataset that has only one linkage phase for every LG. For the QTL mapping procedure, the settings for the CIM program were forward and backward stepwise regression, a window size of 10, 2.0 cM walking speed, and a *p*-value < 0.05 after 1000 permutations. QTL interval was defined for the chromosomal region having a LOD score above the permutation threshold.

We searched for regions of colocalized QTLs which include all QTLs mapped for FRH, NDVI, SE, and FD using LS means and BLUP. This was done to search for chromosomal regions containing putative pleiotropic genes controlling multiple traits. Colocalized QTLs are those with the overlapped QTL intervals.

## Results

### Frequency distribution of the phenotypic traits

**Fig. 1:**
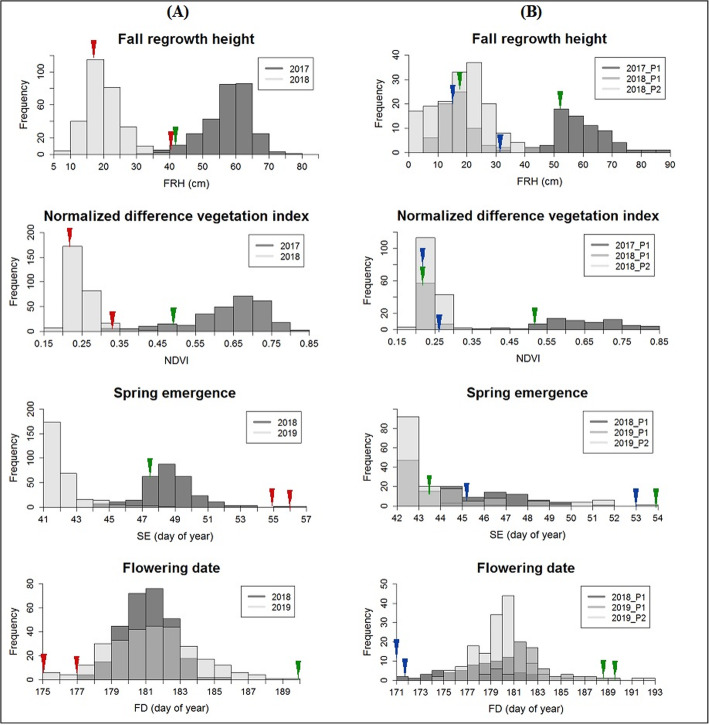
Frequency distribution of the phenotypic traits in AB (column A) and BV (column B) populations for two years of field evaluation. *P1* The first portion of the BV population that was planted in April 2017; *P2* The second portion of the BV population that was planted in May 2018; Red triangle = AP13; Green triangle = B6; Blue triangle = VS16

**Fig. 2:**
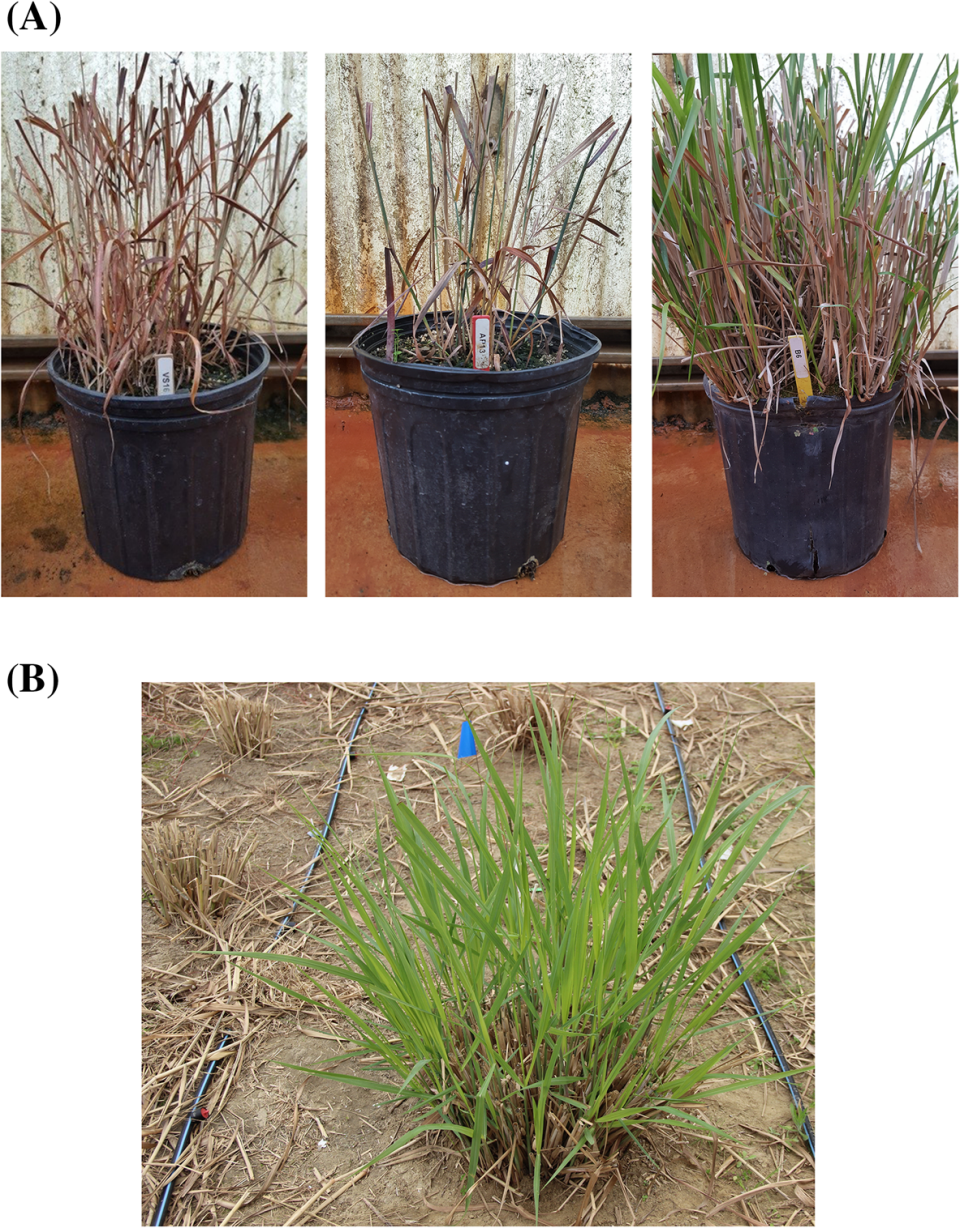
**a** Parent plants in the greenhouse during winter (February 2020) showed dormant VS16 and AP13 and non-dormant B6; **b** Growth of B6 plant in Tifton, GA during winter (January 2020), six weeks after clipping in November showed that the plant is still actively growing

FRH and NDVI were the first data collected in the first year of planting. Both populations had a similar range of distribution for FRH and NDVI (Fig. 1). B6 had higher FRH and NDVI than AP13 and VS16. For FRH and NDVI collected in fall 2018, both population distributions were skewed to the right, indicating lower population FRH and NDVI values. The reason for the shift in values was because we clipped the plants on 31st August in 2017 and on September 21st in 2018. Thus, most of the plants were already dormant or progressing toward dormancy in fall 2018, and hence the lower FRH and NDVI values. There was no B6 value in the AB plot for 2018 because the plant was growing poorly in spring 2018 and eventually died in fall 2018. In the BV plot in the fall of 2018 B6 value was similar to VS16. B6 had earlier emergence than AP13 but later emergence than VS16 in spring 2018. In spring 2019, B6 had earlier emergence than VS16. We think the reason for B6 year to year variation is most likely due to its intolerance to cold temperature. B6 grows throughout the winter in the greenhouse where the temperature is warmer while AP13 and VS16 go dormant (Fig. 2a). Both populations had earlier emergence in spring 2019 than 2018, indicating that the environment in early spring 2019 was more conducible for growth. For the flowering date, both populations have about the same range of distribution in 2018 and 2019. B6 flowered later than AP13 and VS16, which suggests that B6 completed its growth later than the other two parents.

### Analysis of variance

There was a significant genotype effect on all traits in AB, BV planting date 1, and BV planting 2, and a significant year effect for FRH, NDVI, and SE in AB; FRH, NDVI, SE, and FD for BV (Table 1). A significant genotype by year effect was observed for NDVI in AB, FRH, NDVI, SE, and FD for BV planting date 1. Because of this significant interaction, QTL mapping was done using LS means for each year and BLUP for trait values across years.

**Table 1:** Mean squares and significance of fall regrowth height (FRH), normalized difference vegetation index (NDVI), spring emergence (SE), and flowering date (FD) in two switchgrass F1 populations

Source of variation	FRH		NDVI		SE		FD	
	df	MS	df	MS	df	MS	df	MS
AP13 x B6								
Genotype (G)	284	150.25**	284	0.021**	284	9**	284	21**
Year (Y)	1	589,950.00**	1	63.400**	1	16372**	1	45^ns^
G x Y	284	70.12^ns^	284	0.013**	284	5^ns^	284	4^ns^
Rep (Year)	4	6800.37**	4	0.301**	4	13*	4	130**
Residuals	1117	68.08	1118	0.009	1114	5	1115	6
B6 x VS16 (Planting 1)^a^								
Genotype (G)	65	220.92**	65	0.020**	65	3*	65	30**
Year (Y)	1	159,930.00**	1	14.779**	1	990**	1	1567*
G x Y	65	87.86**	65	0.013**	65	4**	65	6*
Rep (Year)	4	215.87**	4	0.016^ns^	4	15**	4	76**
Residuals	237	51.30	236	0.008	236	2	230	4
B6 x VS16 (Planting 2)^b^								
Genotype	160	255.89**	160	0.002**	160	15**	160	19**
Rep	2	242.32^ns^	2	0.004**	2	5^ns^	2	76**
Residuals	315	93.83	314	0.001	302	8	302	8

^a^The first group of BV population that was planted in April 2017; ^b^The second group of BV population that was planted in May 2018; *MS* mean square **p < 0.01; *p < 0.05; ^ns^ not significant

### Broad-sense heritability (H^2^)

There were medium ranges of H^2^ for FRH (0.54–0.64) and NDVI (0.30–0.61), a small to medium range of H^2^ for SE (0.13–0.56), and a high range of H^2^ for FD (0.61–0.88) across AB, BV planting date 1, and BV planting date 2 (Table 2). In most cases, the highest H^2^ was observed for FD, followed by FRH, NDVI, and SE.

**Table 2:** Broad-sense heritability and variance component for fall regrowth height (FRH), normalized difference vegetation index (NDVI), spring emergence (SE), and flowering date (FD) in two switchgrass F1 populations

Variance component	FRH	NDVI	SE	FD
AP13 x B6				
V_G_	14.1634	0.0015	1.1137	3.1996
V_GY_	0.9358	0.0016	0.0547	0.0000
V_E_	68.0466	0.0088	5.1854	5.5072
H^2^	0.5453	0.3933	0.5554	0.7771
B6 x VS16 (Planting 1)^A^				
V_G_	23.9085	0.0012	0.1453	7.1659
V_GY_	17.5303	0.0029	1.0892	0.5953
V_E_	51.4146	0.0078	2.4063	4.0671
H^2^	0.5797	0.2997	0.1332	0.8802
B6 x VS16 (Planting 2)^B^				
V_G_	54.6427	0.0004	2.1842	4.0238
V_E_	93.9269	0.0007	8.5569	7.6346
H^2^	0.6357	0.6068	0.4337	0.6126

*V*_*G*_ Genotypic variance; *V*_*GY*_ Genotype by year variance; *V*_*E*_ Error variance; *H*^*2*^ Broad-sense heritability; ^A^The first portion of BV population that was planted in April 2017; ^B^The second portion of BV population that was planted in May 2018

### Correlation between traits

A similar trend of correlation was observed for AB and BV populations (Table 3). Biomass weight was positively correlated with FRH, NDVI, and FD, while negatively correlated with SE, which means higher biomass weight is correlated with lower dormancy level, earlier spring emergence, and later flowering. FD had the lowest correlation with other traits; it was not correlated with biomass in BV, and with SE in AB and BV.

**Table 3:**
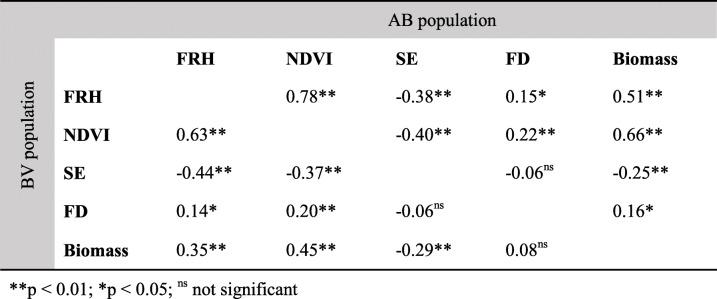
Pearson correlation coefficients between winter dormancy related traits and dry biomass weight for AB (upper diagonal) and BV (lower diagonal) populations

### Significant QTLs

Using 2772 and 3766 SNP markers for AB and BV population, respectively, for QTL mapping, we identified 16, 14, 12, and 20 QTLs for FRH, NDVI, SE, and FD, respectively, mapped in both populations and years using LS means (Supplementary Table 1–4). For FRH QTLs, 3 were mapped in AB and 13 in BV. For NDVI QTLs, 4 were mapped in AB and 10 from BV. For SE QTLs, 7 were mapped in AB and 5 in BV. For FD QTLs, 11 were mapped in AB and 9 in BV. A higher total number of QTLs were mapped in the BV population; these could be due to the separate mapping done for two subsets of the BV population. Another reason could be due to a higher genetic variance between B6 and VS16, leading to more QTLs contributing to phenotypic expression in F1 progenies. To have a meaningful comparison, QTL numbers are compared using BLUP QTLs. We discovered 9, 6, 11, and 14 significant BLUP QTLs for FRH, NDVI, SE, and FD, respectively (Supplementary Table 1–4). A higher number of BLUP QTLs were observed in the BV population for FRH (6 QTLs) and NDVI (4), while a higher BLUP QTLs were found for SE in the AB population (6 QTLs), and an equal amount of QTLs found in both populations (7 QTLs each).

**Fig. 3:**
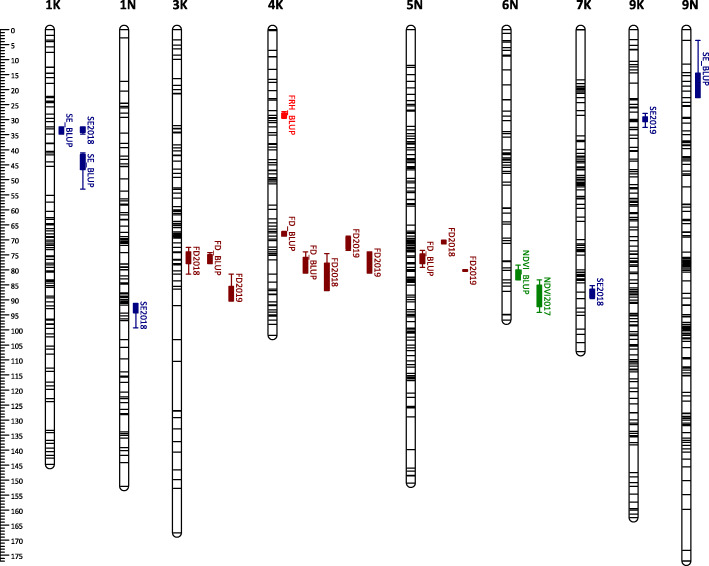
QTL position and colocalization in the AP13 map of the AB population. QTLs are positioned at the right side of each LG; solid bars and whiskers on one or both ends represent coverage at LOD drop interval of 1.0 and 2.0, respectively. QTLs were mapped using LS means for each year and the BLUP value, and labeled with the trait they are associated with followed by year (LS means) or BLUP suffixes

**Fig. 4:**
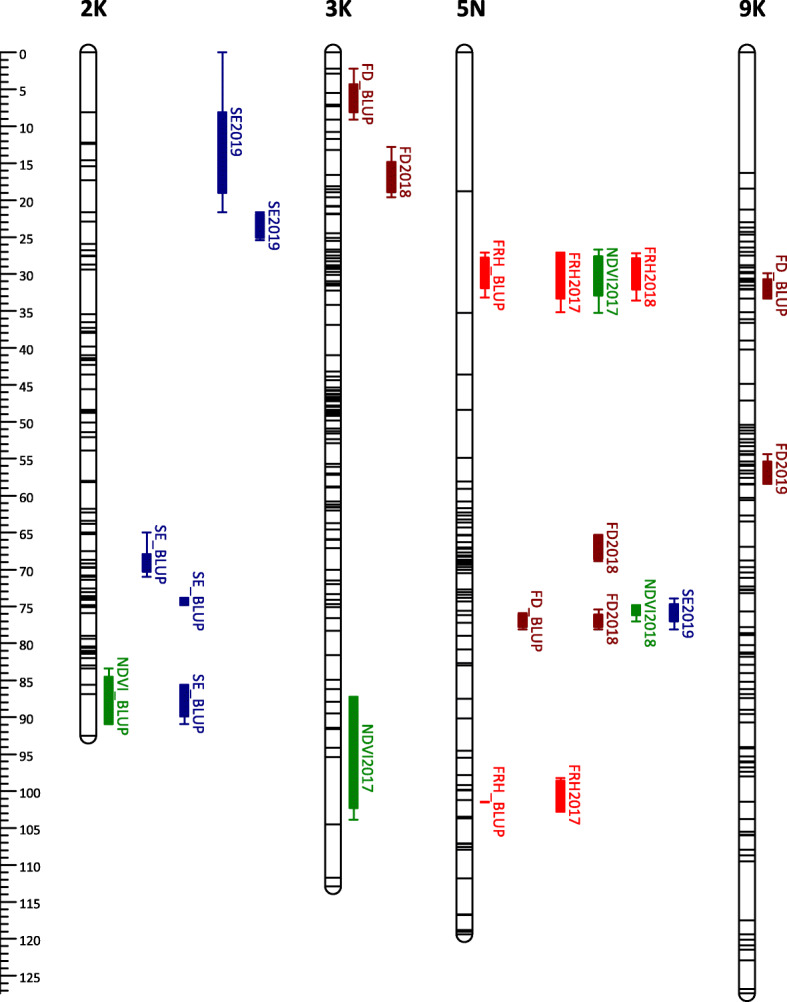
QTL position and colocalization in the B6 map of the AB population. QTLs are positioned at the right side of each LG; solid bars and whiskers on one or both ends represent coverage at LOD drop interval of 1.0 and 2.0, respectively. QTLs were mapped using LS means for each year and the BLUP value, and labeled with the trait they are associated with followed by year (LS means) or BLUP suffixes

**Fig. 5:**
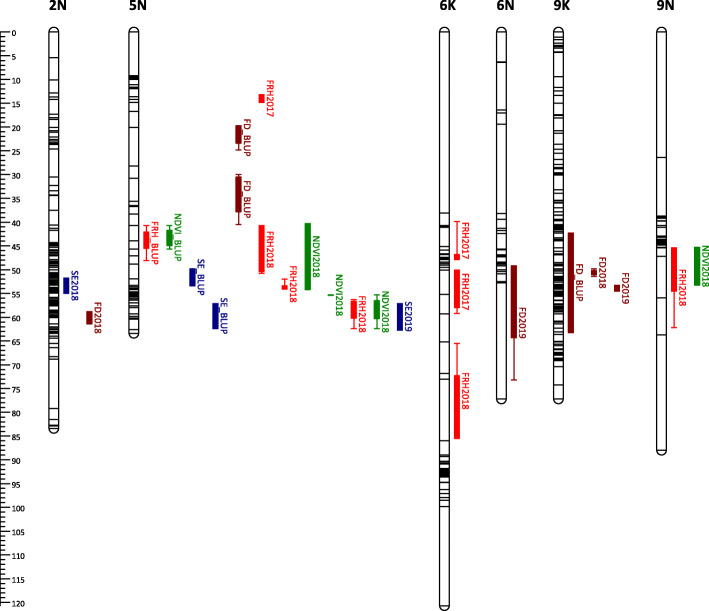
QTL position and colocalization in the B6 map of the BV population. QTLs are positioned at the right side of each LG; solid bars and whiskers on one or both ends represent coverage at LOD drop interval of 1.0 and 2.0, respectively. QTLs were mapped using LS means for each year in each planting date, and the BLUP value, and labeled with the trait they are associated with followed by year (LS means) or BLUP suffixes

**Fig. 6:**
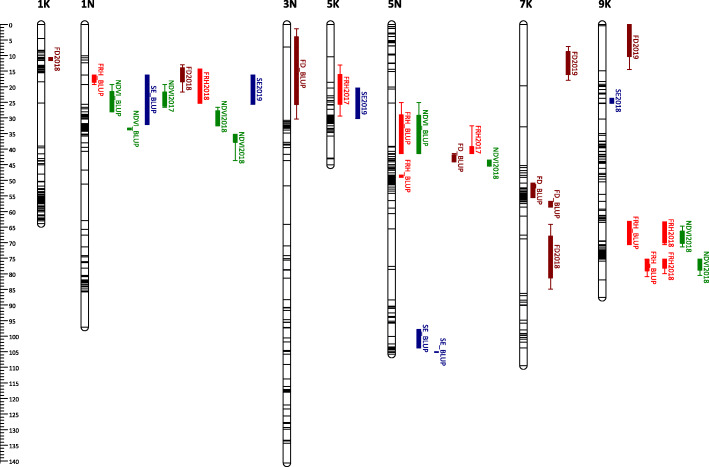
QTL position and colocalization in the VS16 map of the BV population. QTLs are positioned at the right side of each LG; solid bars and whiskers on one or both ends represent coverage at LOD drop interval of 1.0 and 2.0, respectively. QTLs were mapped using LS means for each year in each planting date, and the BLUP value, and labeled with the trait they are associated with followed by year (LS means) or BLUP suffixes

Some of the QTLs mapped with BLUP values overlapped with those mapped using LS means. We identified 7, 2, 2, and 4 BLUP QTLs for FRH, NDVI, SE, and FD, which are redundant to LS means QTLs (Supplementary Table 5). Adding all QTLs mapped with LS means and unique BLUP QTLs, we have a total of 18 QTLs for FRH, 18 QTLs for NDVI, 21 QTLs for SE, and 30 QTLs for FD (Figs. 3, 4, 5 and 6). For FRH, the range of percentage of variance explained (PVE) by each QTL is 4.21–23.27%, for NDVI this is 4.47–24.06%, for SE the range is 4.35–32.77%, and for FD the range is 4.61–29.74%. FRH QTLs were mapped in LG 5 N and 4 K in the AB population; LG 1 N, 5 K, 5 N, 6 K, 9 K, and 9 N in the BV population. NDVI QTLs were found in LG 2 K, 3 K, 5 N, and 6 N in the AB population; LG 1 N, 5 N, 9 K, and 9 N in the BV population. SE QTLs were mapped in LG 1 K, 1 N, 2 K, 5 N, 7 K, 9 K, 9 N in the AB population; LG 1 N, 2 N, 5 K, 5 N, and 9 K in the BV population. FD QTLs were found in LG 3 K, 4 K, 5 N, and 9 K in the AB population; LG 1 K, 1 N, 2 N, 3 N, 5 N, 6 N, 7 K, and 9 K in the BV population.

### QTL colocalization

Combining all QTLs mapped using LS means and BLUP (redundant BLUP QTLs were not included), we observed a total of 16 QTL colocalization regions within all parental maps in both populations (Fig. 3, 4, 5 and 6, Supplementary Table 6). There are three chromosomal regions with colocalization of 3 QTLs. The first region is in LG 5 N of B6.AB map; the three QTLs found here are mapped for SE2019 (marker AB6919), NDVI2018 (marker AB6919), and FD2018 (marker AB8070). The NDVI and FD QTLs in this region have positive additive effects (α) while SE QTL has a negative α. This is a possible indication that the transmission of this chromosomal region can reduce the dormancy level by having a higher magnitude of plant greenness in the fall, earlier dormancy break as indicated by early spring emergence, and later flowering/maturity.

The second region with 3 colocalized QTLs is in LG 5 N of the B6.BV map. The QTLs found in this region were mapped for FRH2018 (marker BV17469), NDVI2018 (marker BV17469), and SE2019 (marker BV17309). The FRH and NDVI QTLs have negative α while the SE QTL has a positive α. Since this region is associated with a higher dormancy level (positive α for FRH and NDVI) and later dormancy break (negative α), we can potentially use the opposite marker genotype to screen the plants with the opposite trait direction, i.e. lower dormancy and early emergence. A similar condition is observed in the third colocalization region with QTLs mapped for FRH2018 (marker BV7842), SE2019 (marker BV7842), and FD2018 (marker BV7842); the FRH and FD QTLs have negative α while the SE QTL has a positive α.

FRH and NDVI are two traits that shared the most number of colocalized QTLs regions with 8 regions in total. The second highest is NDVI and SE with 4 regions. NDVI has the most number of overlapped QTL regions with other traits with 14 regions in total, while FD had the least number of colocalized regions with other traits with 5 colocalized regions. In contrast, FD has the most number of the same QTL mapped across years with 3 QTLs in total.

## Discussion

Initiation of winter dormancy and its release, vegetative growth, and development of reproductive organs occur sequentially following plant perception of environmental stimuli like changes in day length and temperature. These stimuli trigger the expressions of genes that regulate the growth of different plant structures such as shoots, stems, flowers, rhizomes, and roots. The identification of the genomic regions associated with seasonal growth changes offers the possibility of tapping the genetic potential of switchgrass to produce higher biomass yield through the extension of the growth period.

Through our observation of most switchgrass growth in the field, it peaks during the long days and warm temperature of the late spring (May–June), starts flowering when sensing the gradual decrease in daylengths in mid-summer (July to early August), and finally undergoes senescence (September–October) and becomes dormant (November to January) when days are short and cold in the winter. Identifying the QTLs associated with these growth changes would enable using them as markers for selecting progenies with delayed dormancy and flowering, and with early spring emergence. In our study, we used FRH and NDVI as indicators of dormancy status, SE for dormancy exit, and FD as an indication for the plant reaching the end of its growth cycle.

It is important to understand the mechanism underlying stand persistence of switchgrass so that the changes made to the growth cycle will not negatively affect its survival. Switchgrass persistence is particularly impacted by C and N translocation from the shoots to crowns, rhizomes, and roots where lower mobilization can result in plant death [29]. This can happen in the case of low tillering and disruption of senescence [29]. Sarath et al. [29] suggested a longer growth period of the southern germplasms compared to the northern germplasms when planted in a northern environment. However the plants do not have a defined period of senescence before winter dormancy, and hence do not fully cycle C and N. As a consequence, the non-adapted germplasm suffers from the loss of structures crucial to perenniality [29]. In southern locations where winter usually starts at a later date and generally less cold than northern locations, planting non-dormant or semi-dormant cultivars can potentially increase biomass yield, as long as the plant can properly translocate nutrients to the belowground storage organs before winter starts. In addition to death caused by poor C and N translocation, switchgrass deaths can also be caused by severe frost, suggesting a non-adaptation of cellular mechanisms to freezing and lack of cold acclimation [29].

We did not phenotype cold tolerance in this study but recognize the importance of this trait for warm-season plant survival under low temperatures. We observed some mortality of the B6 parent that might be a result of intolerance to freezing temperature as it originated from a Florida collection. The B6 parent has been observed to persist and grow very well through winter at the more southern location Tifton, where winter is milder than the location where we carried out this study (Fig. 2b). It has also been observed to grow during winter in the greenhouse where other parents (AP13 and VS16) were gone completely dormant, typical of a nondormant genotype (Fig. 2a). AP13 is a lowland genotype that is known to have a high biomass yield while VS16 is an upland genotype with lower yield but better tolerance to cold. Both populations exhibited a continuous bell-shaped distribution for all four phenotypes, suggesting they are quantitative traits controlled by many genes. This explains the large number of QTL that were successfully mapped for all traits.

Heritability was calculated to see if traits can be passed on to progenies without being affected by the environmental changes. Although we did not test the progenies in different locations, the different years bring enough environmental differences because of the variability in rainfall, temperature, nutrient status, etc. We also clipped the plants at a later date in 2018, thus the FRH and NDVI values were smaller in 2018 compared to 2017 for both populations. The difference in the year impact was shown by the significant year and genotype by year interaction for the majority of the traits. In our study we found FD to have the highest heritability, followed by FRH, NDVI, and SE.

Correlation of traits with dry biomass yield was calculated to test the hypothesis that an extended growth period leads to more biomass accumulation in these two F1 populations. We have previously found a positive correlation in a switchgrass diversity panel consisting of 17 lowland, 5 intermediate, and 14 upland accessions [32]. In the current study biomass weight was confirmed again to be positively correlated with FRH and NDVI, while negatively correlated with SE, for both populations. The positive correlation between biomass weight and FD was weak for the AB population and non-significant for the BV population. This means biomass weight increases with the increase in growth and plant greenness in the fall, and earlier plant emergence. Later flowering date increases the biomass yield only to a smaller magnitude in AB, which indicates the disadvantage of using the trait for indirect selection of high biomass yield, particularly in the southern region with longer growing season and switchgrass is observed to flower in early summer. We found a high correlation between FRH and NDVI (0.78 r in AB and 0.63 r in BV), intermediate correlations between FRH and SE (− 0.38 r in AB and − 0.44 r in BV) and between NDVI and SE (− 0.40 r in AB and − 0.37 r in BV), and low or non-significant correlations between FD and other traits. For highly correlated traits, we think a similar gene pathway or gene action is involved in controlling the expression of the traits.

We have successfully mapped 18 QTLs for FRH, 18 QTLs for NDVI, 21 QTLs for SE, and 30 QTLs for FD. We found a higher number of QTL in the BV population, specifically in the VS16 map. This can be explained by the higher genetic divergence of the parents used for the cross [43]. We found that the BV population contained more polymorphic markers and that the parents have the largest genetic distance. Since the parents were originally adapted to different latitudes they should have more variants contributing to traits segregation in the progenies. In the case of linkage mapping in a pseudo-testcross population (with separate parental genetic maps), the variation within the parental genome is captured through the trait-marker association. On the other hand for the AB population, since both parents originally adapted to southern latitudes, they theoretically have less genetic diversity, thus fewer variants contributing to trait segregation. Another reason might be the high rates of segregation distortion of alleles in the progenies of the AB population [43], which may have resulted in a lower percentage of mappable markers and possible dropout of alleles associated with the traits understudied [44, 45].

We found some QTLs reoccurring in the second year of evaluation (using LS means); the reoccurrence of these QTLs suggests the high heritability of the genes linked to those QTLs. There are 3 common FD QTLs and 1 common FRH QTLs mapped for both years. As both FD (0.62–0.88 H^2^) and FRH (0.55–0.64 H^2^) have high trait heritability, this explains why common QTLs were found for these two traits. The markers located near the QTL positions can potentially be used for progeny screening as they give more confidence in the trait expression.

QTLs colocalization indicates either pleiotropic gene action or different genes that are closely linked. For the latter, the association between different traits can be broken after a few cycles of recombination. For pleiotropic gene action, we can utilize the QTL to simultaneously select for multiple favorable traits such as lower dormancy level, early emergence, and later flowering/maturity. Colocalization of QTLs could also explain why certain traits are highly correlated with each other. We found 8 colocalized regions with FRH and NDVI QTLs, and these two traits were highly correlated with each other (0.78 r in AB and 0.63 r in BV). FD has the lowest number of colocalized regions with other traits, as also shown by its low correlation with other traits.

There are several QTL mapping studies done in switchgrass, these include mapping of QTL for spring green-up, flowering time, developmental traits, and biomass weight [46–53]. Dong et al. [52] conducted QTL mapping for reproductive maturity and found the QTL in LG 1a, 2b, 3a, 3b, 7a, 8b, and 9a. In our study, we also mapped flowering QTL in LG 1 N (1a), 3 K (3a), 3 N (3b), and 7 K (7a). Milano et al. [54] found QTL for flowering date in LG 2 K, 4 K, 5 K, 5 N, 9 K, and 9 N. In our study, we also found flowering QTL in LG 4 K, 5 N, and 9 K. Tornqvist et al. [47] did QTL mapping of heading and anthesis dates and mapped the QTL in LG 2 K, 2 N, 3 K, 4 K, 4 N, 7 N, 8 N, and 9 K. We also mapped the flowering QTL in LG 2 N, 3 K, 4 K, and 9 K. Ali et al. [46] performed QTL mapping of spring green-up and days to flower. They identified spring green-up QTL in LG 1 K, 1 N, 2 N, 3 K, 3 N, 4 N, 5 K, 6 K, 6 N, 8 K, 8 N, 9 K, and 9 N. Days to flower QTL were mapped in 1 K, 1 N, 2 K, 2 N, 3 K, 4 K, 5 K, 5 N, 6 K, 6 N, 7 K, 7 N, 8 N, and 9 N. In our study, we also found the spring emergence QTL in LG 1 K, 1 N, 2 N, 5 K, 9 K, and 9 N, and flowering date QTL in LG 1 K, 1 N, 2 N, 3 K, 4 K, 5 N, 6 N, and 7 K. Poudel et al. [55] found QTL associated with tiller numbers phenotyped during spring emergence after undergoing staged freezing treatment in LG 1 K, 5 K, 5 N, and 9 K. We mapped the spring emergence QTL in the same LGs.

Flowering time was the focus of many QTL mapping studies due to its potential in extending the growth period and increase biomass yield. However, our study showed that biomass yield was correlated more with winter dormancy than flowering date, suggesting that winter dormancy QTL can be used to screen for plants with potential high biomass yield to be grown in the southern region. To date, there are no published QTL mapping studies on winter dormancy besides the few transcriptomic studies aiming to identify gene pathways and processes involved during senescence and dormancy [56–58]. Poudel et al. [59] developed genomic selection models to predict southern germplasms’ winter survival in northern regions. They found higher prediction accuracy with better genetic relatedness between the training and validation populations. The study did not phenotype winter dormancy per se but the survival rate of plants after freezing winter seasons which is a function of adequate senescence, cold acclimation, and cold tolerance. Our study is the first report on QTL (and their genomic regions) associated with winter dormancy and the trait implication in the accumulation of more biomass through the extension of the growth period. The markers found within the QTL interval are potential genomic resources that can be used in marker-assisted breeding programs. These markers merit more investigations in future work to validate the status and level of association with the traits in other populations with different genetic backgrounds.

## Conclusion

Identifying the genomic regions associated with switchgrass seasonal growth changes is important for the genetic manipulation and extension of switchgrass growing season. Planting non-dormant or semi-dormant switchgrass in southern locations with mild winters is a potential strategy for the accumulation of more biomass. We have successfully identified 18 QTLs for FRH, 18 QTLs for NDVI, 21 QTLs for SE, and 30 QTLs for FD, with a higher number of QTLs mapped in the BV (lowland x upland) population. Breeding superior cultivars can be done through the incorporation of alleles that are associated with improved traits. Using markers linked to these traits enabled the screening of progenies in the early growth stage. In future work, we will validate the effect of the significant QTLs in a population with different genetic backgrounds.

## Supplementary Information

**Additional file 1.**

## Data Availability

All data generated or analyzed during this study are included in this published article (and its Supplementary Information files). Variant files and their metadata are publicly available at Figshare.com (10.6084/m9.figshare.13120271).

## Abbreviations

FRH: Fall regrowth height
NDVI: Normalized difference vegetation index
SE: Spring emergence
FD: Flowering date
AB: AP13 x B6 population
BV: B6 x VS16 population
QTL: Quantitative trait loci
LS means: Least square means
BLUP: Best Linear Unbiased Predictor
LG: Linkage group
H^2^: Broad-sense heritability
r: Correlation coefficient
LOD: Logarithm of odds
cM: centiMorgan
PVE: Percentage of trait variance
