# Supplementary material for: QTL mapping of winter dormancy and associated traits in two switchgrass pseudo-F1 populations: lowland x lowland and lowland x upland
Source: BMC Plant Biol. 2020 Nov 30;20:537. doi: 10.1186/s12870-020-02714-8 (PMC7708163; doi:10.1186/s12870-020-02714-8)
Supplement: Supplementary file 1 — Additional file 1. [file 12870_2020_2714_MOESM1_ESM.docx]

| **Supplementary table 1** QTL found significantly associated with fall regrowth height (FRH) in two switchgrass F1 populations for two years of field evaluation and with BLUP values across years | | | | | | | | | | |
| --- | --- | --- | --- | --- | --- | --- | --- | --- | --- | --- |
| Year | Map-Population | LG | Marker at LOD peak | Position in the genetic map (cM) | Position in the *P. virgatum* V4.1 (bp) | LOD | PVE (%) | Additive (cm) | Left flanking marker | Right flanking marker |
| 2017 | B6.AB | 5N | AB7387 | 28.86 | 16080695 | 4.73 | 6.22 | 3.72 | AB7387 | AB7387 |
|  | B6.AB | 5N | AB14427 | 101.44 | 23465 (Scaffold 859) | 4.64 | 6.08 | 3.71 | AB7134 | AB8135 |
|  | B6.BV.P1 | 5N | BV15640 | 14.24 | 2581322 | 3.18 | 12.28 | -6.84 | BV15640 | BV15640 |
|  | B6.BV.P1 | 6K | BV17760 | 47.36 | 4450457 | 3.84 | 15.03 | 7.20 | BV17760 | BV30761 |
|  | B6.BV.P1 | 6K | BV17729 | 55.19 | 2608727 | 3.70 | 14.72 | 7.18 | BV17700 | BV17729 |
|  | VS16.BV.P1 | 5K | BV13227 | 22.89 | 7103662 | 3.55 | 13.77 | -7.07 | BV13227 | BV13350 |
|  | VS16.BV.P1 | 5N | BV15983 | 40.68 | 16530569 | 5.56 | 23.27 | -18.71 | BV15974 | BV16032 |
| 2018 | B6.AB | 5N | AB7387 | 28.86 | 16080695 | 6.80 | 9.39 | 3.36 | AB8078 | AB7373 |
|  | B6.BV.P1 | 5N | BV16525 | 45.68 | 48627372 | 3.96 | 16.77 | -4.97 | BV28750 | BV16498 |
|  | B6.BV.P1 | 5N | BV16808 | 53.58 | 73181217 | 3.31 | 14.33 | -4.61 | BV16808 | BV30676 |
|  | B6.BV.P1 | 6K | BV17912 | 73.02 | 9778083 | 4.58 | 21.48 | 5.89 | BV18296 | BV17912 |
|  | B6.BV.P1 | 9N | BV28082 | 47.17 | 18482004 | 3.13 | 14.88 | 4.45 | BV28082 | BV28082 |
|  | B6.BV.P2 | 5N | BV17469 | 58.05 | 94244589 | 5.07 | 11.45 | -6.63 | BV17590 | BV17375 |
|  | VS16.BV.P2 | 1N | BV7842 | 16.19 | 103630976 (2N) | 4.85 | 12.19 | -6.59 | BV7842 | BV2083 |
|  | VS16.BV.P2 | 9K | BV26161 | 63.51 | 42160044 | 4.39 | 9.85 | -5.94 | BV26161 | BV27023 |
|  | VS16.BV.P2 | 9K | BV26581 | 75.92 | 62574526 | 4.90 | 9.96 | -6.00 | BV26632 | BV26581 |
| BLUP | AP13.AB | 4K | AB5171 | 28.62 | 11825144 | 4.75 | 7.32 | -1.91 | AB5171 | AB5205 |
|  | B6.AB | 5N | AB7387 | 28.86 | 16080695 | 9.13 | 12.57 | 1.77 | AB8078 | AB7373 |
|  | B6.AB | 5N | AB14426 | 101.38 | 23448 (Scaffold 859) | 3.39 | 4.21 | 1.05 | AB14426 | AB14427 |
|  | B6.BV | 5N | BV16393 | 43.89 | 36643284 | 7.92 | 13.66 | -4.27 | BV15934 | BV16843 |
|  | VS16.BV | 1N | BV7842 | 16.19 | 103630976 (2N) | 6.64 | 11.40 | -3.84 | BV1894 | BV2253 |
|  | VS16.BV | 5N | BV15983 | 40.68 | 16530569 | 4.01 | 5.77 | -2.77 | BV15974 | BV16098 |
|  | VS16.BV | 5N | BV16488 | 48.52 | 46597975 | 3.70 | 5.43 | -2.71 | BV16488 | BV16488 |
|  | VS16.BV | 9K | BV27070 | 69.45 | 77072403 | 3.51 | 5.67 | -2.72 | BV27070 | BV27023 |
|  | VS16.BV | 9K | BV26581 | 75.92 | 62574526 | 4.32 | 6.23 | -2.85 | BV26581 | BV26581 |
| Map-population, *AP13.AB* AP13 map of the AB population, *B6.AB* B6 map of the AB population, *B6.BV.P1* B6 map of the first subset of the BV population, *B6.BV.P2* B6 map of the second subset of the BV population, *VS16.BV.P1* VS16 map of the first subset of the BV population, *VS16.BV.P2* VS16 map of the second subset of the BV population; *LG* linkage group; *cM* Centimorgan position; *LOD* logarithm of odds score; *PVE* percentage of trait variance explained by the QTL | | | | | | | | | | |

| **Supplementary table 2** QTL found significantly associated with normalized difference vegetation index (NDVI) in two switchgrass F1 populations for two years of field evaluation and with BLUP values across years | | | | | | | | | | |
| --- | --- | --- | --- | --- | --- | --- | --- | --- | --- | --- |
| Year | Map-Population | LG | Marker at LOD peak | Position in the genetic map (cM) | Position in the *P. virgatum* V4.1 (bp) | LOD | PVE (%) | Additive (cm) | Left flanking marker | Right flanking marker |
| 2017 | AP13.AB | 6N | AB9342 | 87.18 | 76759767 | 4.55 | 6.45 | -0.054 | AB9281 | AB9342 |
|  | B6.AB | 3K | AB4513 | 94.11 | 71289693 | 3.14 | 4.47 | -0.044 | AB4513 | AB4513 |
|  | B6.AB | 5N | AB7387 | 28.86 | 16080695 | 5.95 | 8.27 | 0.063 | AB7387 | AB7369 |
|  | VS16.BV.P1 | 1N | BV2253 | 25.60 | 12147769 | 3.95 | 18.43 | -0.092 | BV2253 | BV2253 |
| 2018 | B6.AB | 5N | AB6919 | 75.36 | 100027529 (5K) | 5.78 | 8.33 | 0.023 | AB7732 | AB4422 |
|  | B6.BV.P1 | 5N | BV16498 | 48.83 | 47917380 | 5.65 | 24.06 | -0.024 | BV28750 | BV30676 |
|  | B6.BV.P1 | 5N | BV30682 | 55.37 | 20398 (Scaffold 1589) | 4.90 | 20.65 | -0.023 | BV30682 | BV30682 |
|  | B6.BV.P1 | 9N | BV28082 | 47.17 | 18482004 | 4.18 | 17.93 | 0.020 | BV28067 | BV28082 |
|  | B6.BV.P2 | 5N | BV17469 | 58.05 | 94244589 | 5.72 | 12.39 | -0.019 | BV17153 | BV17375 |
|  | VS16.BV.P2 | 1N | BV2509 | 29.77 | 32302068 | 3.71 | 7.41 | -0.014 | BV24860 | BV2403 |
|  | VS16.BV.P2 | 1N | BV2351 | 35.77 | 17408827 | 4.34 | 8.65 | -0.015 | BV2351 | BV2351 |
|  | VS16.BV.P2 | 5N | BV16155 | 44.90 | 24461350 | 3.42 | 6.95 | -0.013 | BV16119 | BV16154 |
|  | VS16.BV.P2 | 9K | BV27022 | 69.62 | 76454585 | 5.63 | 11.33 | -0.017 | BV27070 | BV26833 |
|  | VS16.BV.P2 | 9K | BV26581 | 75.92 | 62574526 | 4.01 | 8.27 | -0.015 | BV26807 | BV26581 |
| BLUP | AP13.AB | 6N | AB9304 | 80.16 | 73988736 | 3.59 | 5.52 | -0.015 | AB9281 | AB9342 |
|  | B6.AB | 2K | AB2582 | 86.90 | 89588059 | 3.58 | 5.12 | -0.015 | AB2582 | AB2582 |
|  | B6.BV | 5N | BV16393 | 43.89 | 36643284 | 6.44 | 10.96 | -0.012 | BV15934 | BV16843 |
|  | VS16.BV | 1N | BV2253 | 25.60 | 12147769 | 4.07 | 6.53 | -0.009 | BV2253 | BV2459 |
|  | VS16.BV | 1N | BV2504 | 33.36 | 31905859 | 3.35 | 5.40 | -0.008 | BV2657 | BV2593 |
|  | VS16.BV | 5N | BV15974 | 39.13 | 16315596 | 3.86 | 6.49 | -0.009 | BV15974 | BV16098 |
| Map-population, *AP13.AB* AP13 map of the AB population, *B6.AB* B6 map of the AB population, *B6.BV.P1* B6 map of the first subset of the BV population, *B6.BV.P2* B6 map of the second subset of the BV population, *VS16.BV.P1* VS16 map of the first subset of the BV population, *VS16.BV.P2* VS16 map of the second subset of the BV population; *LG* linkage group; *cM* Centimorgan position; *LOD* logarithm of odds score; *PVE* percentage of trait variance explained by the QTL | | | | | | | | | | |

| **Supplementary Table 3.** QTL found significantly associated with spring emergence (SE) in two switchgrass F1 populations for two years of field evaluation and with BLUP values across years | | | | | | | | | | |
| --- | --- | --- | --- | --- | --- | --- | --- | --- | --- | --- |
| Year | Map-Population | LG | Marker at LOD peak | Position in the genetic map (cM) | Position in the *P. virgatum* V4.1 (bp) | LOD | PVE (%) | Additive (cm) | Left flanking marker | Right flanking marker |
| 2018 | AP13.AB | 1K | AB148 | 32.97 | 11827854 | 3.46 | 4.55 | -0.77 | AB148 | AB148 |
|  | AP13.AB | 1N | AB1512 | 91.66 | 81667979 | 4.20 | 5.52 | 0.82 | AB1512 | AB1512 |
|  | AP13.AB | 7K | AB9971 | 87.44 | 69558759 | 3.48 | 4.62 | -0.75 | AB9971 | AB9971 |
|  | B6.BV.P1 | 2N | BV6999 | 53.24 | 75358244 | 3.96 | 17.12 | -1.79 | BV6937 | BV7179 |
|  | VS16.BV.P1 | 9K | BV25526 | 25.25 | 8705146 | 6.80 | 32.77 | -3.08 | BV25473 | BV25526 |
| 2019 | AP13.AB | 9K | AB11882 | 29.92 | 5889294 | 2.95 | 4.59 | -0.60 | AB11882 | AB11882 |
|  | B6.AB | 2K | AB14332 | 14.59 | 40918 (Scaffold 228) | 3.71 | 5.32 | -0.65 | AB1810 | AB1862 |
|  | B6.AB | 2K | AB1921 | 22.91 | 10576481 | 3.60 | 5.21 | -0.65 | AB1921 | AB1921 |
|  | B6.AB | 5N | AB6919 | 75.36 | 100027529 (5K) | 3.05 | 4.35 | -0.59 | AB6919 | AB6919 |
|  | B6.BV.P2 | 5N | BV17309 | 60.18 | 90805385 | 3.41 | 7.67 | 1.38 | BV16821 | BV17375 |
|  | VS16.BV.P1 | 5K | BV13381 | 27.16 | 10082045 | 3.46 | 15.55 | 0.43 | BV13227 | BV13480 |
|  | VS16.BV.P2 | 1N | BV7842 | 16.19 | 103630976 (2N) | 3.53 | 7.44 | 1.32 | BV7842 | BV2253 |
| BLUP | AP13.AB | 1K | AB148 | 32.97 | 11827854 | 3.42 | 4.61 | -0.30 | AB148 | AB148 |
|  | AP13.AB | 1K | AB217 | 43.95 | 15589691 | 3.33 | 4.53 | -0.30 | AB217 | AB217 |
|  | AP13.AB | 9N | AB12976 | 20.49 | 1897966 | 3.35 | 4.58 | 0.30 | AB12976 | AB12989 |
|  | B6.AB | 2K | AB2223 | 68.66 | 67332134 | 3.65 | 5.32 | 0.37 | AB2223 | AB2221 |
|  | B6.AB | 2K | AB3334 | 73.97 | 79474643 (2N) | 4.70 | 6.89 | 0.41 | AB14404 | AB6461 |
|  | B6.AB | 2K | AB2582 | 86.90 | 89588059 | 5.57 | 8.64 | 0.44 | AB2355 | AB2582 |
|  | B6.BV | 5N | BV17153 | 51.94 | 86558020 | 3.78 | 6.43 | 0.49 | BV17153 | BV17157 |
|  | B6.BV | 5N | BV17345 | 60.35 | 91721591 | 3.36 | 5.74 | 0.46 | BV16821 | BV17345 |
|  | VS16.BV | 1N | BV24860 | 29.21 | 53481584 (8N) | 4.19 | 6.74 | 0.49 | BV2083 | BV2470 |
|  | VS16.BV | 5N | BV17586 | 100.07 | 99158450 | 3.73 | 5.96 | 0.46 | BV17586 | BV17598 |
|  | VS16.BV | 5N | BV17612 | 105.11 | 99763337 | 3.30 | 5.30 | 0.43 | BV17626 | BV17624 |
| Map-population, *AP13.AB* AP13 map of the AB population, *B6.AB* B6 map of the AB population, *B6.BV.P1* B6 map of the first subset of the BV population, *B6.BV.P2* B6 map of the second subset of the BV population, *VS16.BV.P1* VS16 map of the first subset of the BV population, *VS16.BV.P2* VS16 map of the second subset of the BV population; *LG* linkage group; *cM* Centimorgan position; *LOD* logarithm of odds score; *PVE* percentage of trait variance explained by the QTL | | | | | | | | | | |

| **Supplementary Table 4.**  QTL found significantly associated with flowering date (FD) in two switchgrass F1 populations for two years of field evaluation and with BLUP values across years | | | | | | | | | | |
| --- | --- | --- | --- | --- | --- | --- | --- | --- | --- | --- |
| Year | Map-Population | LG | Marker at LOD peak | Position in the genetic map (cM) | Position in the *P. virgatum* V4.1 (bp) | LOD | PVE (%) | Additive (cm) | Left flanking marker | Right flanking marker |
| 2018 | AP13.AB | 3K | AB4202 | 76.51 | 30783935 | 4.79 | 6.22 | -0.77 | AB4090 | AB4107 |
|  | AP13.AB | 4K | AB5480 | 81.41 | 55439856 | 4.31 | 5.33 | 0.67 | AB5480 | AB5940 |
|  | AP13.AB | 5N | AB7559 | 70.63 | 31374185 | 6.72 | 8.63 | -0.87 | AB7575 | AB7749 |
|  | B6.AB | 3K | AB3868 | 18.48 | 11447245 | 3.52 | 4.61 | 0.63 | AB3868 | AB3868 |
|  | B6.AB | 5N | AB7680 | 65.50 | 55319361 | 3.44 | 4.68 | 0.61 | AB7680 | AB7742 |
|  | B6.AB | 5N | AB8070 | 77.04 | 93748125 | 5.57 | 7.50 | 0.78 | AB8005 | AB3733 |
|  | B6.BV.P1 | 2N | BV7292 | 59.77 | 85792456 | 3.54 | 11.26 | -1.94 | BV6885 | BV7409 |
|  | B6.BV.P1 | 9K | BV26182 | 50.29 | 42415733 | 5.60 | 19.10 | -2.58 | BV26158 | BV26512 |
|  | VS16.BV.P1 | 1K | BV258 | 10.92 | 7044151 | 3.59 | 13.40 | 2.06 | BV240 | BV200 |
|  | VS16.BV.P1 | 1N | BV7842 | 16.19 | 103630976 (2N) | 4.33 | 16.70 | -2.36 | BV7842 | BV7842 |
|  | VS16.BV.P1 | 7K | BV21199 | 68.81 | 63927787 | 5.29 | 23.58 | 3.01 | BV21136 | BV21199 |
| 2019 | AP13.AB | 3K | AB13389 | 86.43 | 25300682 (9N) | 5.74 | 7.33 | -1.48 | AB4202 | AB4399 |
|  | AP13.AB | 4K | AB5379 | 69.72 | 42363274 | 4.72 | 5.89 | 1.27 | AB5392 | AB5422 |
|  | AP13.AB | 4K | AB5460 - AB5480 | 76.682 - 81.411 | 51420666 - 55439856 | 3.45 | 4.75 | 1.14 | AB5460 | AB5480 |
|  | AP13.AB | 5N | AB7749 | 80.17 | 72050340 | 7.13 | 9.05 | -1.62 | AB7561 | AB7859 |
|  | B6.AB | 9K | AB12144 | 56.03 | 21935868 | 3.32 | 4.78 | -1.11 | AB12144 | AB12144 |
|  | B6.BV.P1 | 9K | BV26345 | 53.87 | 50875028 | 4.47 | 23.10 | -2.64 | BV26171 | BV26398 |
|  | B6.BV.P2 | 6N | BV19770 | 52.80 | 68878323 | 3.34 | 7.22 | 1.44 | BV19846 | BV19770 |
|  | VS16.BV.P1 | 7K | BV21805 | 0.00 | 9418370 (7N) | 4.50 | 29.74 | -2.71 | BV21805 | BV21805 |
|  | VS16.BV.P1 | 9K | BV25364 | 0.00 | 5373579 | 3.40 | 14.29 | 1.83 | BV25364 | BV25363 |
| BLUP | AP13.AB | 3K | AB4202 | 76.51 | 30783935 | 5.15 | 6.58 | -0.81 | AB4076 | AB13389 |
|  | AP13.AB | 4K | AB5365 | 67.69 | 41228006 | 3.98 | 4.93 | 0.66 | AB5267 | AB5365 |
|  | AP13.AB | 4K | AB5460 - AB5480 | 76.682 - 81.411 | 51420666 - 55439856 | 4.25 | 5.57 | 0.70 | AB5460 | AB5480 |
|  | AP13.AB | 5N | AB7733 | 77.62 | 71127139 | 6.80 | 8.78 | -0.89 | AB7561 | AB7859 |
|  | B6.AB | 3K | AB4634 | 7.29 | 5387960 (3N) | 3.53 | 4.73 | 0.65 | AB4634 | AB4634 |
|  | B6.AB | 5N | AB8070 | 77.04 | 93748125 | 4.67 | 6.25 | 0.73 | AB8071 | AB7360 |
|  | B6.AB | 9K | AB12002 | 32.15 | 10306279 | 3.73 | 4.94 | -0.67 | AB11837 | AB12002 |
|  | B6.BV | 5N | BV15694 | 20.10 | 5715852 | 4.19 | 7.31 | -1.04 | BV15694 | BV15694 |
|  | B6.BV | 5N | BV15847 - BV15934 | 30.781 - 35.579 | 9838597 - 15161360 | 6.22 | 11.04 | -1.28 | BV15847 | BV16393 |
|  | B6.BV | 9K | BV26380 | 54.79 | 51505776 | 6.06 | 9.71 | -1.18 | BV25922 | BV26560 |
|  | VS16.BV | 3N | BV24226 | 7.17 | 5074954 (8N) | 3.15 | 5.20 | 0.96 | BV24226 | BV24226 |
|  | VS16.BV | 5N | BV16098 | 43.46 | 21862533 | 3.16 | 5.24 | -0.87 | BV16098 | BV16098 |
|  | VS16.BV | 7K | BV20802 | 54.38 | 54349204 | 3.64 | 6.11 | 0.94 | BV20735 | BV20873 |
|  | VS16.BV | 7K | BV20829 | 57.51 | 54510476 | 4.17 | 6.96 | 1.00 | BV20931 | BV20780 |
| Map-population, *AP13.AB* AP13 map of the AB population, *B6.AB* B6 map of the AB population, *B6.BV.P1* B6 map of the first subset of the BV population, *B6.BV.P2* B6 map of the second subset of the BV population, *VS16.BV.P1* VS16 map of the first subset of the BV population, *VS16.BV.P2* VS16 map of the second subset of the BV population; *LG* linkage group; *cM* Centimorgan position; *LOD* logarithm of odds score; *PVE* percentage of trait variance explained by the QTL | | | | | | | | | | |

| **Supplementary Table 5.** The same QTLs mapped using LS means and BLUP values | | | | | | | | | |
| --- | --- | --- | --- | --- | --- | --- | --- | --- | --- |
| No. | Trait | Map-Population | LG | Marker at LOD peak | Position in the genetic map (cM) | Position in the *P. virgatum* V4.1 (bp) | LOD | Additive | PVE  (%) |
| 1 | SE_BLUP | AP13.AB | 1K | AB148 | 32.97 | 11827854 | 3.42 | -0.30 | 4.61 |
|  | SE2018 | AP13.AB | 1K | AB148 | 32.97 | 11827854 | 3.46 | -0.77 | 4.55 |
| 2 | FD2018 | AP13.AB | 3K | AB4202 | 76.51 | 30783935 | 4.79 | -0.77 | 6.22 |
|  | FD_BLUP | AP13.AB | 3K | AB4202 | 76.51 | 30783935 | 5.15 | -0.81 | 6.58 |
|  | FD2019 | AP13.AB | 3K | AB13389 | 86.43 | 25300682 (9N) | 5.74 | -1.48 | 7.33 |
| 3 | FD2019 | AP13.AB | 4K | AB5460 - AB5480 | 76.68 - 81.41 | 51420666 - 55439856 | 3.45 | 1.14 | 4.75 |
|  | FD_BLUP | AP13.AB | 4K | AB5460 - AB5480 | 76.68 - 81.41 | 51420666 - 55439856 | 4.25 | 0.70 | 5.57 |
|  | FD2018 | AP13.AB | 4K | AB5480 | 81.41 | 55439856 | 4.31 | 0.67 | 5.33 |
| 4 | FRH_BLUP | B6.AB | 5N | AB7387 | 28.86 | 16080695 | 9.13 | 1.77 | 12.57 |
|  | FRH2017 | B6.AB | 5N | AB7387 | 28.86 | 16080695 | 4.73 | 3.72 | 6.22 |
|  | FRH2018 | B6.AB | 5N | AB7387 | 28.86 | 16080695 | 6.80 | 3.36 | 9.39 |
| 5 | FD_BLUP | B6.AB | 5N | AB8070 | 77.04 | 93748125 | 4.67 | 0.73 | 6.25 |
|  | FD2018 | B6.AB | 5N | AB8070 | 77.04 | 93748125 | 5.57 | 0.78 | 7.50 |
| 6 | FRH_BLUP | B6.AB | 5N | AB14426 | 101.38 | 23448 (Scaffold 859) | 3.39 | 1.05 | 4.21 |
|  | FRH2017 | B6.AB | 5N | AB14427 | 101.44 | 23465 (Scaffold 859) | 4.64 | 3.71 | 6.08 |
| 7 | FRH_BLUP | B6.BV | 5N | BV16393 | 43.89 | 36643284 | 7.92 | -4.27 | 13.66 |
|  | FRH2018 | B6.BV.P1 | 5N | BV16525 | 45.68 | 48627372 | 3.96 | -4.97 | 16.77 |
| 8 | NDVI_BLUP | B6.BV | 5N | BV16393 | 43.89 | 36643284 | 6.44 | -0.01 | 10.96 |
|  | NDVI2018 | B6.BV.P1 | 5N | BV16498 | 48.83 | 47917380 | 5.65 | -0.02 | 24.06 |
| 9 | SE2019 | B6.BV.P2 | 5N | BV17309 | 60.18 | 90805385 | 3.41 | 1.38 | 7.67 |
|  | SE_BLUP | B6.BV | 5N | BV17345 | 60.35 | 91721591 | 3.36 | 0.46 | 5.74 |
| 10 | FD2018 | B6.BV.P1 | 9K | BV26182 | 50.29 | 42415733 | 5.60 | -2.58 | 19.10 |
|  | FD2019 | B6.BV.P1 | 9K | BV26345 | 53.87 | 50875028 | 4.47 | -2.64 | 23.10 |
|  | FD_BLUP | B6.BV | 9K | BV26380 | 54.79 | 51505776 | 6.06 | -1.18 | 9.71 |
| 11 | FRH2018 | VS16.BV.P2 | 1N | BV7842 | 16.19 | 103630976 (2N) | 4.85 | -6.59 | 12.19 |
|  | FRH_BLUP | VS16.BV | 1N | BV7842 | 16.19 | 103630976 (2N) | 6.64 | -3.84 | 11.40 |
| 12 | NDVI_BLUP | VS16.BV | 1N | BV2253 | 25.60 | 12147769 | 4.07 | -0.01 | 6.53 |
|  | NDVI2017 | VS16.BV.P1 | 1N | BV2253 | 25.60 | 12147769 | 3.95 | -0.09 | 18.43 |
| 13 | FRH2017 | VS16.BV.P1 | 5N | BV15983 | 40.68 | 16530569 | 5.56 | -18.71 | 23.27 |
|  | FRH_BLUP | VS16.BV | 5N | BV15983 | 40.68 | 16530569 | 4.01 | -2.77 | 5.77 |
| 14 | FRH2018 | VS16.BV.P2 | 9K | BV26161 | 63.51 | 42160044 | 4.39 | -5.94 | 9.85 |
|  | FRH_BLUP | VS16.BV | 9K | BV27070 | 69.45 | 77072403 | 3.51 | -2.72 | 5.67 |
| 15 | FRH_BLUP | VS16.BV | 9K | BV26581 | 75.92 | 62574526 | 4.32 | -2.85 | 6.23 |
|  | FRH2018 | VS16.BV.P2 | 9K | BV26581 | 75.92 | 62574526 | 4.90 | -6.00 | 9.96 |
| Map-population, *AP13.AB* AP13 map of the AB population, *B6.AB* B6 map of the AB population, *B6.BV.P1* B6 map of the first subset of the BV population, *B6.BV.P2* B6 map of the second subset of the BV population, *VS16.BV.P1* VS16 map of the first subset of the BV population, *VS16.BV.P2* VS16 map of the second subset of the BV population; *LG* linkage group; *cM* Centimorgan position; *LOD* logarithm of odds score; *PVE* percentage of trait variance explained by the QTL | | | | | | | | | |

| **Supplementary Table 6.** Colocalized QTLs within the same linkage map | | | | | | | | | | |
| --- | --- | --- | --- | --- | --- | --- | --- | --- | --- | --- |
| No. | Trait | Map-Population | LG | Marker at LOD peak | Position in the genetic map (cM) | Position in the *P. virgatum* V4.1 (bp) | QTL interval (cM) | LOD | Additive | PVE  (%) |
| 1 | FD2018 | AP13.AB | 3K | AB4202 | 76.51 | 30783935 | 68.79 - 83.76 | 4.79 | -0.77 | 6.22 |
|  | FD2019 | AP13.AB | 3K | AB13389 | 86.43 | 25300682 (9N) | 74.19 - 97.93 | 5.74 | -1.48 | 7.33 |
| 2 | FD2019 | AP13.AB | 4K | AB5460 - AB5480 | 76.68 - 81.41 | 51420666 - 55439856 | 78.69 - 78.69 | 3.45 | 1.14 | 4.75 |
|  | FD2018 | AP13.AB | 4K | AB5480 | 81.41 | 55439856 | 78.69 - 90.67 | 4.31 | 0.67 | 5.33 |
| 3 | NDVI_BLUP | B6.AB | 2K | AB2582 | 86.90 | 89588059 | 86.61 - 88.91 | 3.58 | -0.02 | 5.12 |
|  | SE_BLUP | B6.AB | 2K | AB2582 | 86.90 | 89588059 | 77.9 - 91.91 | 5.57 | 0.44 | 8.64 |
| 4 | FRH2017 | B6.AB | 5N | AB7387 | 28.86 | 16080695 | 28.14 - 32.86 | 4.73 | 3.72 | 6.22 |
|  | NDVI2017 | B6.AB | 5N | AB7387 | 28.86 | 16080695 | 26.14 - 35.16 | 5.95 | 0.06 | 8.27 |
|  | FRH2018 | B6.AB | 5N | AB7387 | 28.86 | 16080695 | 18.5 - 37.74 | 6.80 | 3.36 | 9.39 |
| 5 | SE2019 | B6.AB | 5N | AB6919 | 75.36 | 100027529 (5K) | 75.37 - 75.37 | 3.05 | -0.59 | 4.35 |
|  | NDVI2018 | B6.AB | 5N | AB6919 | 75.36 | 100027529 (5K) | 67.51 - 84.73 | 5.78 | 0.02 | 8.33 |
|  | FD2018 | B6.AB | 5N | AB8070 | 77.04 | 93748125 | 71.59 - 78.79 | 5.57 | 0.78 | 7.50 |
| 6 | FRH2018 | B6.BV.P1 | 5N | BV16525 | 45.68 | 48627372 | 38.28 - 48.84 | 3.96 | -4.97 | 16.77 |
|  | NDVI2018 | B6.BV.P1 | 5N | BV16498 | 48.83 | 47917380 | 40.28 - 54.2 | 5.65 | -0.02 | 24.06 |
| 7 | FRH2018 | B6.BV.P2 | 5N | BV17469 | 58.05 | 94244589 | 22.11 - 62.65 | 5.07 | -6.63 | 11.45 |
|  | NDVI2018 | B6.BV.P2 | 5N | BV17469 | 58.05 | 94244589 | 50.84 - 62.65 | 5.72 | -0.02 | 12.39 |
|  | SE2019 | B6.BV.P2 | 5N | BV17309 | 60.18 | 90805385 | 57.05 - 62.65 | 3.41 | 1.38 | 7.67 |
| 8 | FD2018 | B6.BV.P1 | 9K | BV26182 | 50.29 | 42415733 | 48.55 - 59.32 | 5.60 | -2.58 | 19.10 |
|  | FD2019 | B6.BV.P1 | 9K | BV26345 | 53.87 | 50875028 | 49.83 - 55.61 | 4.47 | -2.64 | 23.10 |
| 9 | FRH2018 | B6.BV.P1 | 9N | BV28082 | 47.17 | 18482004 | 49.18 - 49.18 | 3.13 | 4.45 | 14.88 |
|  | NDVI2018 | B6.BV.P1 | 9N | BV28082 | 47.17 | 18482004 | 45.3 - 53.18 | 4.18 | 0.02 | 17.93 |
| 10 | FRH2018 | VS16.BV.P2 | 1N | BV7842 | 16.19 | 103630976 (2N) | 14.3 - 25.31 | 4.85 | -6.59 | 12.19 |
|  | SE2019 | VS16.BV.P2 | 1N | BV7842 | 16.19 | 103630976 (2N) | 16.2 - 25.61 | 3.53 | 1.32 | 7.44 |
|  | FD2018 | VS16.BV.P1 | 1N | BV7842 | 16.19 | 103630976 (2N) | 14.3 - 18.2 | 4.33 | -2.36 | 16.70 |
| 11 | NDVI2017 | VS16.BV.P1 | 1N | BV2253 | 25.60 | 12147769 | 23.31 - 25.61 | 3.95 | -0.09 | 18.43 |
|  | SE_BLUP | VS16.BV | 1N | BV24860 | 29.21 | 53481584 (8N) | 16.2 - 32.13 | 4.19 | 0.49 | 6.74 |
| 12 | FRH2017 | VS16.BV.P1 | 5K | BV13227 | 22.89 | 7103662 | 20.41 - 24.92 | 3.55 | -7.07 | 13.77 |
|  | SE2019 | VS16.BV.P1 | 5K | BV13381 | 27.16 | 10082045 | 20.41 - 30.21 | 3.46 | 0.43 | 15.55 |
| 13 | NDVI_BLUP | VS16.BV | 5N | BV15974 | 39.13 | 16315596 | 31.18 - 43.47 | 3.86 | -0.01 | 6.49 |
|  | FRH2017 | VS16.BV.P1 | 5N | BV15983 | 40.68 | 16530569 | 33.18 - 42.39 | 5.56 | -18.71 | 23.27 |
| 14 | FD_BLUP | VS16.BV | 5N | BV16098 | 43.46 | 21862533 | 43.47 - 43.47 | 3.16 | -0.87 | 5.24 |
|  | NDVI2018 | VS16.BV.P2 | 5N | BV16155 | 44.90 | 24461350 | 43.47 - 45.54 | 3.42 | -0.01 | 6.95 |
| 15 | FRH2018 | VS16.BV.P2 | 9K | BV26161 | 63.51 | 42160044 | 63.52 - 69.63 | 4.39 | -5.94 | 9.85 |
|  | NDVI2018 | VS16.BV.P2 | 9K | BV27022 | 69.62 | 76454585 | 65.52 - 72.72 | 5.63 | -0.02 | 11.33 |
| 16 | FRH2018 | VS16.BV.P2 | 9K | BV26581 | 75.92 | 62574526 | 74.66 - 77.93 | 4.90 | -6.00 | 9.96 |
|  | NDVI2018 | VS16.BV.P2 | 9K | BV26581 | 75.92 | 62574526 | 74.06 - 77.93 | 4.01 | -0.01 | 8.27 |
| Map-population, *AP13.AB* AP13 map of the AB population, *B6.AB* B6 map of the AB population, *B6.BV.P1* B6 map of the first subset of the BV population, *B6.BV.P2* B6 map of the second subset of the BV population, *VS16.BV.P1* VS16 map of the first subset of the BV population, *VS16.BV.P2* VS16 map of the second subset of the BV population; *LG* linkage group; *cM* Centimorgan position; *LOD* logarithm of odds score; *PVE* percentage of trait variance explained by the QTL | | | | | | | | | | |
